# Fast Screening and Identification of Illegal Adulterated Glucocorticoids in Dietary Supplements and Herbal Products Using UHPLC-QTOF-MS With All-Ion Fragmentation Acquisition Combined With Characteristic Fragment Ion List Classification

**DOI:** 10.3389/fchem.2021.785475

**Published:** 2021-12-10

**Authors:** Ying Xue, Yanghao Sheng, Jue Wang, Qi Huang, Fengyu Zhang, Ying Wen, Shao Liu, Yueping Jiang

**Affiliations:** ^1^ Department of Pharmacy, Xiangya Hospital, Central South University, Changsha, China; ^2^ National Clinical Research Center for Geriatric Disorders, Institute for Rational and Safe Medication Practices, Xiangya Hospital, Central South University, Changsha, China

**Keywords:** UHPLC-QTOF-MS, all ion fragmentation, dietary supplements, herbal products, fragment ion classification, illegal glucocorticoids

## Abstract

Ultra-high-performance liquid chromatography coupled with quadrupole time-of-flight mass spectrometry (UHPLC-QTOF-MS) with all-ion fragmentation (AIF) acquisition was established for an identification and quantification of illegal adulterated glucocorticoids in dietary supplements and herbal products. Next, a novel method called characteristic fragment ion list classification (CFILC) was developed for a fast screening of adulterated compounds. CFILC could provide the characteristic ions comprehensively and completely through direct extract from the MS^2^ library instead of finding them manually. This is time-saving and provides fast screening results with a high confidence level by filtering of a pre-calculated threshold of similarity scores for illegal adulterants that are not included in the library as well as for new emerging structural analogs. The obtained results demonstrated the great qualitative and quantitative strength of this approach, providing a promising and powerful method for a routine fast screening of illegal adulterated glucocorticoids.

## Introduction

In recent years, dietary supplements and herbal products have become popular because they are considered safer than synthetic drugs and free of side effects. The dietary supplements and herbal products industry in China have developed rapidly over the past decades. However, some industries illegally add synthetic drugs to improve short-term therapeutic effects, potentially leading to serious health consequences. The demand for a rapid screening of illegal adulterants in dietary supplements and herbal products by clinicians in Chinese hospitals continues to rise.

Glucocorticoids (GCs) include not only steroid hormones secreted by the adrenal cortex but also active synthetic analogs. They are highly effective drugs commonly used for the treatment of allergies, asthma, and autoimmune diseases, due to their ability of reducing inflammation and suppressing allergic reactions and immune system response (Barnes and Adcock, 2009). However, they also have potential side effects including osteoporosis, allergic contact dermatitis, and permanent skin atrophy (Schacke et al., 2002). The risk of these side effects increases when they are used without medical supervision. In addition, laboratory analysis has found that some herbal products and healthy foods contain GCs (Park et al., 2016;Fung and Linn, 2017). A 67-year-old female patient with idiopathic thrombocytopenic purpura had consumed an herbal supplement called “Herbal Health Joint care” for her frozen shoulder and joint pain, which was adulterated with betamethasone-17-valerate (Fung and Linn, 2017). Therefore, a rapid and reliable method for the screening and quantification of GCs is required.

Various methods have been reported for the determination of GCs (Delahaut et al., 1997; Reig et al., 2006; Haneef et al., 2013; Golubovic et al., 2015; Zhou et al., 2016; Laforest et al., 2019; Protti et al., 2020; Wang et al., 2020; Li et al., 2021; Luque-Córdoba and Priego-Capote, 2021), such as gas chromatography-mass spectrometry (GC-MS) (Delahaut et al., 1997), high-performance liquid chromatography (HPLC) (Reig et al., 2006), and liquid chromatography-mass spectrometry (LC–MS) (Haneef et al., 2013; Golubovic et al., 2015; Zhou et al., 2016; Laforest et al., 2019; Protti et al., 2020; Wang et al., 2020; Li et al., 2021; Luque-Córdoba and Priego-Capote, 2021). Among them, liquid chromatography in combination with triple-quadrupole mass spectrometric detection (LC-QQQ MS) dominates the field of GC determination (Haneef et al., 2013; Golubovic et al., 2015; Laforest et al., 2019; Protti et al., 2020; Wang et al., 2020), due to its high sensitivity and selectivity. However, this technology has certain limitations, and one of the most important is that the target compound must be known in advance because the transitions must be preselected. Thus, this method would not be feasible for detecting compounds not included in the library, such as new emerging undeclared chemicals. High-resolution mass spectrometry (HRMS), such as hybrid time of flight (TOF) (Thoren et al., 2016; Whitman and Lynch, 2019; Luque-Córdoba and Priego-Capote, 2021) or Orbitrap (Zhou et al., 2016), has currently gained increasing popularity in compound screening, identification, and quantification because it allows the identification of compounds that are not included in the library or are unexpected, making it possible to overcome this challenge. Despite this advantage, the discovery of compounds that are not included in the library as well as new emerging undeclared chemicals from large MS data is an intricate and time-consuming task. Recently, some compounds with similar activities that have a certain structural commonality, extracted common ion chromatograms (ECICs) based on common fragments and specific fragmentation of adulterants with similar structure, have been used for a rapid screening of known and unknown illegal adulterants (Kim et al., 2017; Ki et al., 2019). However, this method also has some limitations: 1) the common ions used for fast screening were found by manual search according to the collision-induced dissociation (CID) fragmentation pattern and thus may not be complete, resulting in a small number of common ions. This aspect in turn leads to the inability of ECICs to cover most of the compounds in the library. Most of these compounds in the library have only one common ion for fast screening (Kim et al., 2017; Ki et al., 2019), potentially increasing false positive and false negative errors in screening unknown analogs or illegal adulterants outside the library. 2) The common ions found by manual search is time-consuming and labor-intensive. Therefore, a novel method called Characteristic Fragment Ion List Classification (CFILC) was developed to solve the issues mentioned above. This method uses key and assistance ions directly extracted from the MS^2^ library and a filter in form of Jaccard similarity score thresholding to reduce false-positive and false-negative errors. Moreover, the entire method is automatically operated in the R environment, which is time-saving and convenient for different classes of illegal adulterants.

The concept of data-independent acquisition (DIA) was first introduced and applied in proteomics (Venable et al., 2004) and has been used in other fields as well, such as metabolomics (Wang et al., 2019) and general unknown screening (Whitman and Lynch, 2019). On the basis of the width of the isolation window, several representative DIA strategies are available, including all-ion fragmentation (AIF, also called MS^E^) (Geiger et al., 2010; Ventura et al., 2020; Huérfano Barco et al., 2022; Wasito et al., 2022) and sequential window acquisition of all theoretical fragment ion spectra (SWATH) (Gillet et al., 2012). The AIF mode transmits all precursor ions into CID for fragmentation and provides fragment ion information after each full MS scan, enabling qualitative and quantitative results in a single run. On the basis of the simultaneous acquisition of both precursor and product ions, reliable identification of the compounds is feasible. Most importantly, AIF allows the obtainment of sufficient data points of chromatographic peaks to perform a reliable quantification due to the relatively short duty cycle compared to other DIA strategies (Wang et al., 2019).

Therefore, the aim of the current work was the following. 1) The first was to establish an identification and quantification method of GC adulterants in dietary supplements and herbal products by UHPLC-QTOF-MS with the AIF data acquisition strategy. To the best of our knowledge, this is the first time that AIF is applied for the identification and quantification of GC adulterants in dietary supplements and herbal products. 2) The second was to develop a novel screening method called CFILC for a fast screening and implementing screening of GC adulterants in dietary supplements and herbal products.

## Materials and Methods

### Chemicals and Reagents

Prednisolone, prednisone, triamcinolone acetonide, dexamethasone, hydrocortisone, dexamethasone acetate, cortisone acetate, and hydrocortisone acetate were purchased from Energy Chemical Co., Ltd. (Shanghai, China). Clobetasol propionate and beclomethasone dipropionate were purchased from Tokyo Chemical Industry Co., Ltd. (Tokyo, Japan). Fluocinonide and desonide were purchased from Ark Pharm, Inc. (Chicago, IL, USA). Their chemical structures are displayed in Supplementary Figure S1. HPLC-grade acetonitrile was purchased from Merck Inc. (Germany), and HPLC-grade formic acid and ammonium formate were purchased from Sigma Inc. (Marlborough, MA, USA). Purified water was supplied by China Resources C’estbon Beverage Co., Ltd. (Shenzhen, China).

### Standard Solutions

All the individual standard stock solutions were prepared in methanol at a dose of 1.0 mg/ml. An intermediate standard mixture of the reference compounds was prepared by appropriately diluting the individual stock solution in methanol. Matrix-matched working solutions were freshly prepared in blank sample extracts, which were extracted from the commercial products purchased from the local market. All the stock and working solutions were stored at −20°C in the dark.

### Sample Preparation

All samples were processed according to our optimized method. Briefly, a dose of 0.2 g solid sample (for tablets, pills, and capsules) or 0.2 ml liquid sample was extracted by 1 ml methanol and sonicated for 30 min. The sample mixture was centrifuged at 5,000 rpm for 15 min, then filtered through a 0.22-μm microfiltration membrane and subjected to UHPLC-MS analysis. The sample solution was diluted when the concentration was beyond the linear range.

### Instrumentation

A 1290 Infinity ultrahigh-performance liquid chromatography (UHPLC) system coupled to a 6545B quadrupole-time-of-flight (Q-TOF) mass spectrometer (Agilent Technologies, Palo, CA, USA) equipped with a dual Agilent Jet Stream electrospray ionization source (AJS-ESI) was used for all the experiments. Data acquisition and processing were carried out using the MassHunter Workstation software.

Chromatographic separation was performed on a Waters HSS T3 column (100 mm * 2.1 mm, 1.8 μm). The flow rate of the mobile phase was 0.2 ml/min, the column temperature was maintained at 40°C, and the injection volume was 5 μl. The mobile phase was composed of solvent A (0.1% formic acid with 5 mM ammonium formate in water) and solvent B (0.1% formic acid in acetonitrile), and the gradient program was as follows: 30–95% B for 0.0–10.0 min, 95% B for 10.0–15.0 min, and final re-equilibration for 8 min to the initial condition before each injection.

The parameters of the 6545B Q-TOF mass spectrometer, operating in positive mode, were the following: the capillary voltage, nebulizing gas, and fragmentation voltage were set to 3,500 V, 35 psi, and 140 V, respectively. The drying gas temperature and the drying gas flow were set to 350°C and 11 l/min, respectively. The sheath gas temperature and the sheath gas flow were set to 350°C and 10 l/min, respectively. The acquisition was obtained with a mass range of 50–1,000 m*/z* in AIF mode, including two sequential experiments at two alternating collision energies (one full scan at 0 eV, followed by one MS/MS scan at 20 eV). The duty cycle was set to 0.5 s. Data were acquired in centroid mode at the extended dynamic range mode (2 GHz) over a mass range of 0–1,700 m*/z*. The mass calibration of the TOF system was continuously controlled by measuring the protonated reference ions of purine (*m/z* 121.050873) and HP-0921 (*m/z* 922.009798).

### In-House Database Construction

Twelve standard drugs potentially and illegally added to dietary supplements and herbal products were appropriately diluted and characterized to establish the drug in-house database using both the full scan and target-MS/MS modes at a collision energy of 20 eV using the same mobile phase conditions mentioned in *Instrumentation*. The drug information including the molecular formula, retention time, accurate mass of precursor ion, and MS^2^ spectra was stored in MSP format.

### Method Validation

The validation parameters included selectivity, calibration curve, accuracy, precision, matrix effect, recovery, and stability. Quality control (QC) samples at three different concentrations (low, medium, and high) were prepared using 12 compounds to calculate the validation parameters.

#### Selectivity

The selectivity is the ability of an analytical method to distinguish and quantify the analyte in the presence of other components in the matrix, and it was evaluated by determining the level of the interfering components in six individual sources of blank matrix.

#### Calibration Curve

The calibration curve for each standard was used to determine the linearity by the determination coefficient (r^2^), and it was constructed using the peak area (Y-axis) of each standard compound *versus* its corresponding concentration (X-axis). The regression equation was described as *Y = a + bX*, which was used to calculate the concentration of the QCs and samples. The detection limit (LOD) was determined as the lowest concentration giving a signal-to-noise ratio of at least threefold (*S/N >* 3). The lower limit of quantitation (LLOQ) was defined as the lowest concentration of the calibration curve, giving a signal-to-noise ratio of at least 10-fold (*S/N >* 10).

#### Accuracy and Precision

Accuracy describes the closeness mean value of the detected concentration (*C*_det_) obtained using QC samples at three different concentrations (low, medium, and high) to the nominal concentration (*C*_nom_) of the analyte and was from the back-calculated values of the calibration curve. The accuracy was calculated as (C_det_/C_nom_) × 100%. The precision was expressed using the relative standard deviation (RSD), calculated as follows: RSD% = [standard deviation (SD)/C_det_] × 100%. QC samples in six replicates at three different concentrations for 12 compounds were prepared to calculate the intra-day precision in a single day, while the inter-day precision was confirmed within three consecutive days.

#### Matrix Effect

The absolute matrix effect was evaluated by analyzing the response of the analytes prepared in solvent and in the extracted blank matrix (granule and liquid) used at the same concentrations in the three levels (low, medium, and high). The absolute matrix effect was calculated as follows: matrix effect (ME)% = response in matrix-matched standard/response in solvent × 100%.

The relative matrix effect was evaluated using the RSD of the peak area of the analytes in different types of matrices at the same concentration.

#### Recovery

The extraction recovery of GCs was obtained from the response of the analytes added to and extracted from the blank matrices (granule and liquid), compared to the response of the analytes spiked into the solution extracted from the blank matrices.

#### Stability

The stability was assessed using the repeated determination of the standards kept in the autosampler (10°C) for 24 h and evaluated using the RSD of the peak area.

### CFILC Data Process

The CFILC was developed in the R programming environment (ver. 3.6.0), and the script is free available at GitHub (https://github.com/yhshengjy/CFILC). The raw data (*. d) were first converted to the common file format of Reifycs Inc. (*.abf) using the Reifycs ABF converter. After the conversion, the MS-DIAL software (ver. 4.38) (Tsugawa et al., 2015) was used for the detection of the feature, annotation, and spectra deconvolution. Detailed data processing settings of MS-DIAL are shown in Supplementary Table S1. Then, the peak feature table containing both the MS^1^ feature and MS^2^ spectra were imported into CFILC for a fast screening and preliminary identification. The peak features whose similarity score exceeded the threshold were further confirmed in an in-house database or imported into MS-FINDER (Tsugawa et al., 2016) for structural elucidation. The parameters used in MS-FINDER were the one in default, except for the element selection which was “O, F, CL”. MS^2^ fragment ions of GCs are shown in Supplementary Figure S2. Moreover, the fragmentation pattern of GCs is shown in Supplementary Figure S3.

The criteria to identify the drugs were the following: 1) the mass accuracy of the precursor ion should be less than 5 ppm; 2) the deviation tolerance threshold of the retention time was set at 0.2 min; 3) the isotope pattern score was more than 90; 4) more than two specific product ions were correctly confirmed (mass tolerance <10 ppm); and 5) the spectral similarity score (dot product) was more than 800.

## Results and Discussion

### Establishment of the Characteristic Fragment Ions List Classification as a Fast-Screening Method

**FIGURE 1:**
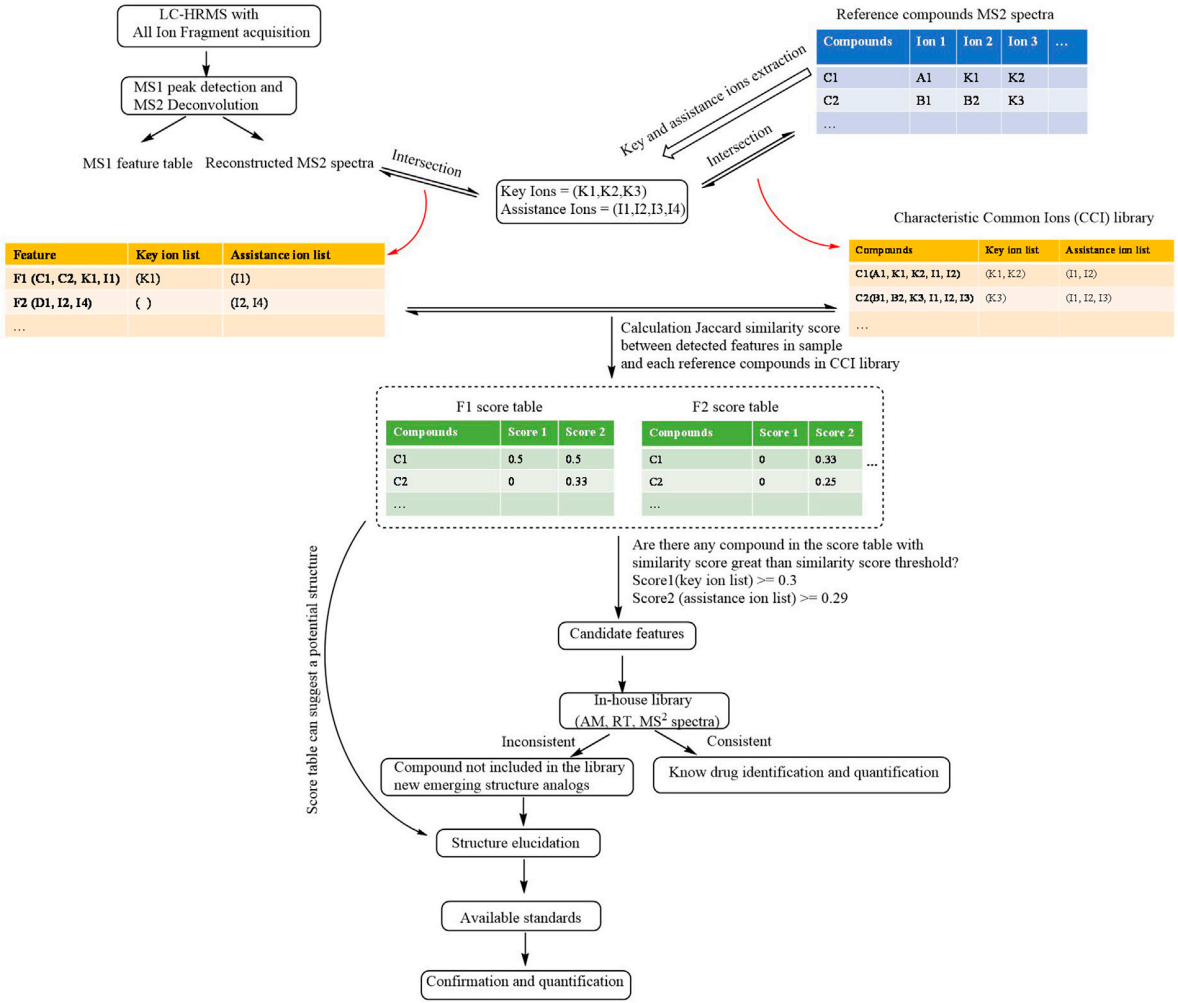
Schematic workflow of CFILC.

The finding of illegal adulterants that are not included in the library and new emerging structure analogs from large MS data is an intricate and time-consuming task. Illegal adulterants and their analogs with a similar structure might present similar fragmentation patterns. Thus, a new method was developed for a fast screening of illegal adulterants according to this theory. Our method could provide comprehensive and complete common ions through the direct extraction from the MS^2^ library instead of finding them manually in ECIC (Kim et al., 2017; Ki et al., 2019). Our method gives results of a high confidence level regarding the fast screening of illegal adulterants that are not included in the library, as well as new emerging structure analogs through the classification of common ions and pre-calculated threshold similarity score. The CFILC workflow is shown in Figure 1.

#### Construction of the Characteristic Common Ions Library and Extraction of Key and Assistance Ions

MS^2^ spectra of compounds with similar structure can be obtained in two manners: 1) MS^2^ spectra (m/z, intensity) of the reference compounds can be exported from publicly available libraries (e.g., mzCloud, METLIN) and commercial libraries; 2) reference compounds can be used to perform UPLC-HRMS with data-dependent acquisition, and product ion information (m/z, intensity) was collected.

The product ions of the reference compounds chosen had at least 5% greater intensity than the most abundant product ions. The key ions represent the common ions of at least half of the compounds in the library. The assistance ions represent the common ions of at least five compounds in the library except those included in the list of the key ions. An individual list of key and assistance ions of each reference compound was obtained in order to construct the CCI library through the intersection of their product ions with their key and assistance ions.

Using the GC adulterants as an example, the MS^2^ spectra (m/z, intensity) of GCs were exported from the Agilent PCDL METLIN database. The product ions with an intensity that is 5% greater than that of the most abundant product ions were chosen for the extraction of key and assistance ions. A total of 14 key ions (*m/z* 225.1274, 263.1430, 147.0804, 223.1117, 171.0804, 237.1274, 251.1430, 239.1430, 161.0961, 173.0961, 121.0648, 185.0961, 211.1117, and 135.0804) and 31 assistance ions were collected after extraction. The CCI library of the GCs was obtained through the intersection of their product ions with their key and assistance ions.

#### Generation of the Individual List of Ions, Calculation of the Similarity score and Classification of the Features

The tables of the MS^1^ peak features and the reconstructed MS^2^ spectrum after MS^1^ peak detection and MS^2^ spectrum deconvolution were imported into the CFILC. The list of individual key and assistance ions of each detected feature which exceed the threshold of MS intensity (e.g., MS^1^ peak height > 10^4^) in the sample was obtained through the intersection of their product ions with their key and assistance ions.

A table of similarity scores listing the similarity score for each detected feature with each reference compound in the CCI library was obtained by calculating the Jaccard similarity score of both the list of key ions (score 1) and the list of assistance ions (score 2) of each reference compound in the CCI library and each feature that was detected in the samples.

Among the scores, if a score exceeded the threshold of the similarity score, the feature could be the one of an illegally added adulterant. The higher the score, the more similar the structure of the detected feature is to that of the corresponding reference compound in the CCI library.

An appropriate similarity score threshold should be set to avoid false-positive and false-negative results. We used the compound in the CCI MS2 library (compared with itself in the library to confirm known adulterants and compared with other compounds in the library to simulate unknown adulterants) and the non-glucocorticoids sample were used as test sample. The non-GC samples include two types of MS^2^ spectra. The first type of MS^2^ spectra is another type of illegal additive, which predominates in the Chinese market. The second type of MS^2^ spectra is blank matrices. The true positive rate (TPR) and false positive rate (FPR) in the test sample were calculated under different combinations by traversing the combination of score 1 and score 2 (stepwise 0.01), and the results revealed that when score 1 was 0.30 and score 2 was 0.29, the maximum Youden index was obtained.

#### Validation of CFILC

Eight compounds not in the CCI library were used for validation. Using alclometasone dipropionate as an example, a table with similarity scores of a feature corresponding to alclometasone dipropionate was obtained through CFILC. Some scores in the score table exceeded the threshold, indicating that the feature could be an illegal adulterant of the class of GCs, and the top 5 scores corresponding to the reference compounds in the CCI library were clobetasol propionate, betamethasone dipropionate, beclomethasone, dexamethasone acetate, and dexamethasone, suggesting a potential structure. Then, the information of the feature was imported into MS-FINDER to elucidate the structure. The possible molecular formulas were C_28_H_37_ClO_7_, C_27_H_31_F_7_O_2_, and C_22_H_39_ClF_2_O_9_ through formula prediction function. Based on the score for formula prediction, C_28_H_37_ClO_7_ was the molecular formula of greatest possibility. Then the feature was analyzed *via* annotation by an *in silico* fragment, and the resulting compounds were beclomethasone dipropionate and alclometasone dipropionate. The feature was identified as alclometasone dipropionate and confirmed by another run using the mode of data-dependent acquisition. Therefore, this method could be used to detect illegal adulterants that are not in the library and to discover unknown illegal adulterants, providing a useful tool for structural elucidation.

The overall 8 compounds were successfully identified except for cortisone acetate, because this compound was very hard to fragment that the intensity of the product ion was lower than the threshold set by the program, leading to a low similarity score. When the threshold of the product ion intensity was decreased, the cortisone acetate was successfully identified. The overall false positive rate was 4.2% based on the threshold of the similarity score. Most of the false positives were in-source fragmentation (especially in GCs) and deconvolute error. The same and adjacent retention time (scan number) of the compound could lead to an incorrect reconstruct MS^2^ spectrum. These issues could be rapidly solved based on scan number, the list of key and assistance ions, and the similarity score and confirmed by another run using the mode of DDA (described in details in *Application in Real Samples*).

#### Application in Real Samples

Five dietary supplements/herbal products submitted by clinical physicians in the past 3 months were considered. Our analysis revealed that two out of five samples contained illegal adulterants. For example, clobetasol propionate was detected in a representative adulterated sample. Firstly, the peak feature table was imported into CFILC for a fast screening and preliminarily identification. A total of 24 features met the requirement of the similarity score threshold. However, the 24 features had a similar retention time (9.89, 9.91, 9.93, and 9.95 min) and scan number (579, 580, 581, and 582) and most of the key and assistance ions in the list and similarity scores were the same, and the top 1 score corresponded to the compound clobetasol propionate. This might indicate that most of these features were false positive results. Then, the 24 features were screened by comparing their accurate mass, retention time, and MS^2^ spectrum with compound in the in-house database, and one of the features was identified as clobetasol propionate. The retention time of the feature was 9.91 min (less than 0.2 min compared to the standard), and the mass error between the measured accurate mass (*m/z* 467.2009) and the accurate mass of clobetasol propionate (*m/z* 467.1995) was 3.0 ppm. The two specific product ions were confirmed, and the spectral similarity score was more than 800. The concentration of clobetasol propionate was 117.76 μg/ml. By combining an accurate mass with retention time, 9 of the 24 features were produced by in-source fragmentation, 5 features were adduct ions of clobetasol propionate, and 9 features were produced by deconvolute error due to the almost similar multiplexed MS^2^ spectrum and the same model peak and confirmed by performing another run using the mode of DDA.

### UHPLC-HRMS Optimization

The optimization of the UHPLC parameters was performed to obtain a short run time, best separation and peak shape, and highest peak intensity for a mixture of 12 compounds. Methanol and acetonitrile were evaluated as the organic mobile solvents, and their use revealed that acetonitrile allowed to obtain a better peak shape and separation of the mixture of the 12 compounds. Furthermore, additional experiments were performed in order to examine different compositions of the aqueous mobile phase, such as formic acid (0.1%), ammonium formate (5 mmol/l), and formic acid (0.1%)–ammonium formate (5 mmol/l). The obtained chromatograms of the compounds showed a considerably better shape of the peaks when a mixture of formic acid–ammonium formate was used. The LC gradient was optimized in order to achieve an efficient separation of the 12 compounds and a run time as short as possible. This procedure using an optimized gradient elution enabled the detection of the 12 compounds within 15 min.

The crucial parameters of AJS-ESI, including capillary voltage, sheath gas temperature, sheath gas flow, and fragmentation voltage were carefully optimized on the full MS scan mode since the MS parameters play an important role on the ion response of the compounds. The values that provided the best response were selected as the optimal values, as described in *Instrumentation*.

**FIGURE 2:**
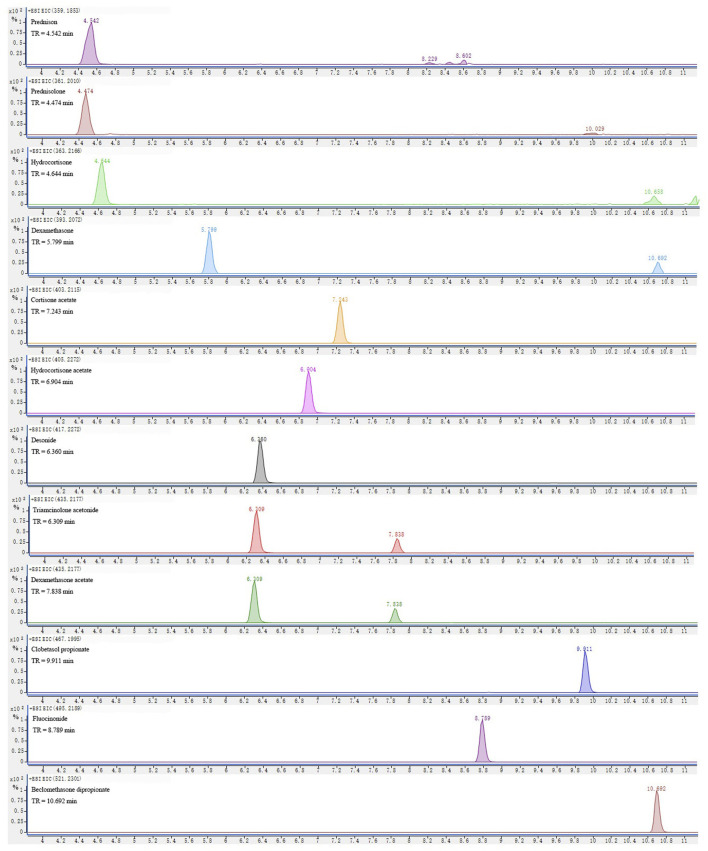
Extracted ion chromatograms (EICs) at ± 10 ppm mass windows for [M + H]^+^ of targeted analytes in a standard solution at 1,000 ng/ml.

The typical extracted ion chromatogram of the 1,000-ng/ml standard mixture spiked with blank matrices is shown in Figure 2. The retention time of compounds varied from 4 to 11 min under the optimized chromatographic conditions (described in *Instrumentation*). The mass of all precursor ions corresponded to the protonated molecule ([M + H]^+^). The mass accuracy was below 5 ppm for all the compounds, suggesting a high level of confidence between the theoretical and experimental mass for all compounds provided by Q-TOF HRMS.

### Method Validation

#### Linearity and Selectivity

The regression equation, coefficient of determination, linear range, LOD, and LLOQ of GCs are listed in Table 1. All the target substances in the QC sample were sensitively detected with a sufficient intensity of the peaks. The LODs of all the examined GCs ranged from 2 to 5 ng/ml, while the LLOQs were from 10 to 20 ng/ml. The linearity was obtained from each calibration curve at six different concentrations in the range from 15.63 to 2,000.00 ng/ml, with *R* ^2^ values greater than 0.9945, thus revealing a good linearity.

**TABLE 1:** Retention time, molecular formula, accurate mass, linearity, and sensitivity of 12 illegal adulterated glucocorticoids obtained by UHPLC-QTOF-MS.

Analyte	RT (min)	Protonated mass [M + H]^+^ (Da)		Mass error (ppm)	Linearity range (ng/ml)	Correlation coefficient (r^2^)	LOD (ng/ml)	LLOQ (ng/ml)
		Theoretical	Experimental					
Prednisolone	4.47	361.2010	361.2002	2.21	15.63–1000.00	0.9966	2.00	10.00
Prednison	4.54	359.1853	359.1850	0.84	15.63–2000.00	0.9972	5.00	15.63
Triamcinolone acetonide	6.35	435.2177	435.2175	0.46	15.63–1000.00	0.9998	2.00	10.00
Dexamethasone	5.80	393.2072	393.2075	−0.76	31.25–2000.00	0.9992	5.00	20.00
Clobetasol propionate	9.91	467.1995	467.1987	1.71	15.63–2000.00	0.9961	2.00	10.00
Beclomethasone dipropionate	10.69	521.2301	521.2289	2.30	15.63–1000.00	0.9989	2.00	10.00
Hydrocortisone	4.64	363.2166	363.2159	1.93	15.63–2000.00	0.9970	5.00	15.63
Fluocinonide	8.79	495.2189	495.2193	−0.81	15.63–1000.00	0.9987	2.00	10.00
Desonide	6.36	417.2272	417.2280	−1.92	15.63–1000.00	0.9977	2.00	10.00
Dexamethasone acetate	7.84	435.2177	435.2196	−4.37	15.63–1000.00	0.9977	5.00	15.63
Cortisone acetate	7.24	403.2115	403.2112	0.74	15.63–1000.00	0.9973	5.00	15.63
Hydrocortisone acetate	6.90	405.2272	405.2275	−0.74	15.63–1000.00	0.9945	2.00	10.00

#### Accuracy and Precision

The results of within-day and between-day accuracy and precision analyses performed at three different concentrations (low, medium, and high) are listed in Table 2. The intra-day and inter-day accuracy ranged from 87.82% to 119.73% and from 89.50% to 109.52%, respectively. The intra- and inter-day precision ranged from 0.93% to 4.92% and from 1.86% to 7.39%, respectively. These results demonstrated that the developed method was reliable, reproducible, and accurate for the quantitative analysis of GCs in dietary supplements and herbal products.

**TABLE 2:** Accuracy, precision, recovery, matrix effect, and stability of 12 illegal adulterated glucocorticoids obtained by UHPLC-QTOF-MS.

Analyte	QC concentration ng/mL	Accuracy (%)		Precision (RSD, %)		Recovery (mean ± SD, %)		Matrix effect (mean ± SD, %)		Stability (RSD, %)
		Intra-day	Inter-day	Intra-day	Inter-day	Solid	Liquid	Solid	Liquid	
Prednisolone	80	119.73	106.19	4.09	3.73	102.70 ± 3.70	101.61 ± 4.66	96.00 ± 4.52	95.28 ± 1.96	5.94
	400	99.26	105.39	2.00	4.09	84.97 ± 3.39	104.16 ± 2.95	102.50 ± 3.53	96.32 ± 2.18	2.67
	1000	102.07	97.96	2.29	3.45	94.64 ± 1.87	105.12 ± 1.45	96.68 ± 2.43	96.30 ± 1.12	3.28
Prednison	80	115.43	107.35	3.18	2.92	89.31 ± 4.40	105.68 ± 3.86	99.27 ± 0.48	93.20 ± 2.10	3.18
	400	93.91	103.53	1.15	3.73	97.28 ± 3.98	104.48 ± 1.72	99.07 ± 1.12	99.42 ± 1.44	2.28
	1000	99.92	98.86	2.45	2.25	93.61 ± 4.46	104.09 ± 1.05	96.56 ± 1.14	96.93 ± 0.84	0.37
Triamcinolone acetonide	80	107.58	98.54	3.05	5.73	96.80 ± 3.43	102.83 ± 4.12	97.64 ± 0.53	97.87 ± 4.13	5.64
	400	107.19	104.54	2.07	5.27	97.71 ± 2.89	102.55 **±** 1.17	99.11 ± 2.34	97.20 ± 2.96	4.05
	1000	95.13	104.04	4.49	4.11	100.36 ± 2.18	103.06 ± 2.45	94.84 ± 1.30	94.83 ± 2.26	3.98
Dexamethasone	80	111.00	108.60	2.41	5.18	97.39 ± 2.74	101.93 ± 4.35	85.84 ± 4.41	96.58 ± 4.52	5.09
	400	91.78	109.52	3.88	4.35	95.09 ± 2.36	100.22 ± 3.76	96.53 ± 3.03	94.90 ± 1.01	2.77
	1000	90.55	105.77	4.92	4.30	95.79 ± 2.07	103.84 ± 0.86	96.64 ± 1.85	97.92 ± 1.90	1.13
Clobetasol propionate	80	89.28	104.32	3.70	7.39	99.12 ± 4.58	95.58 ± 4.20	99.42 ± 6.79	89.47 ± 1.51	2.59
	400	116.35	107.63	1.27	5.18	91.93 ± 3.76	101.02 ± 3.33	98.02 ± 2.92	92.33 ± 2.61	3.99
	1000	94.54	101.64	4.72	4.36	94.16 ± 2.45	104.34 ± 2.04	93.70 ± 2.74	94.77 ± 2.36	3.50
Beclomethasone dipropionate	80	114.84	99.74	1.56	5.57	102.04 ± 5.30	104.27 ± 2.65	109.11 ± 4.13	106.66 ± 2.64	2.12
	400	119.94	99.65	0.93	4.97	89.66 ± 2.99	102.86 ± 1.39	93.24 ± 3.02	89.44 ± 0.43	2.79
	1000	96.90	100.74	4.56	4.20	90.08 ± 4.65	103.03 ± 1.35	96.17 ± 1.32	95.37 ± 0.63	4.30
Hydrocortisone	80	110.85	104.20	3.85	4.02	99.86 ± 3.39	102.55 ± 5.18	96.48 ± 1.53	94.33 ± 0.80	2.54
	400	88.30	104.02	3.22	3.90	95.67 ± 3.58	102.83 ± 2.16	97.17 ± 4.46	95.13 ± 2.35	5.26
	1000	90.64	100.19	4.90	3.62	94.54 ± 6.13	101.90 ± 2.05	91.93 ± 1.27	91.89 ± 0.94	1.40
Fluocinonide	80	102.89	103.85	2.85	3.81	98.71 ± 1.65	104.44 ± 2.44	91.89 ± 1.14	92.14 ± 1.27	1.70
	400	98.86	105.41	1.21	4.32	95.00 ± 1.68	104.18 ± 0.71	97.79 ± 2.38	96.17 ± 0.68	6.40
	1000	91.28	101.49	3.75	4.13	98.01 ± 3.85	104.27 ± 1.17	93.93 ± 1.16	92.18 ± 1.09	2.01
Desonide	80	90.15	102.74	2.32	4.96	95.53 ± 3.69	101.71 ± 1.78	100.11 ± 5.19	93.75 ± 2.23	5.92
	400	109.00	105.04	1.92	4.83	96.08 ± 3.50	102.27 ± 1.30	97.83 ± 3.36	93.63 ± 3.07	4.67
	1000	93.16	101.80	4.81	4.33	92.68 ± 5.91	103.05 ± 0.42	106.22 ± 1.54	94.78 ± 1.13	4.08
Dexamethasone acetate	80	92.95	107.90	3.83	2.61	96.76 ± 3.38	101.06 ± 5.65	90.54 ± 3.07	88.69 ± 3.39	3.15
	400	111.79	93.03	2.16	3.24	97.70 ± 2.34	107.01 ± 2.92	95.94 ± 1.84	94.83 ± 1.91	2.41
	1000	96.35	89.50	2.46	3.72	100.46 ± 2.10	105.31 ± 1.75	97.06 ± 2.61	95.44 ± 2.20	1.46
Cortisone acetate	80	119.86	102.51	3.20	3.11	97.01 ± 2.32	102.44 ± 2.81	96.63 ± 1.62	97.12 ± 1.24	2.16
	400	91.68	98.38	2.76	2.98	92.98 ± 2.20	102.19 ± 1.15	99.70 ± 2.59	99.83 ± 1.86	4.07
	1000	89.62	89.53	2.83	1.86	95.08 ± 4.22	100.04 ± 0.92	91.23 ± 1.61	90.77 ± 1.73	1.42
Hydrocortisone acetate	80	118.55	105.85	2.18	2.90	97.92 ± 3.31	98.20 ± 4.34	102.38 ± 2.15	102.09 ± 2.46	2.97
	400	87.82	103.15	1.27	4.55	95.48 ± 2.50	103.42 ± 1.41	100.45 ± 2.89	102.28 ± 1.52	1.72
	1000	91.16	95.73	3.00	3.93	96.61 ± 2.54	101.90 ± 1.81	90.78 ± 0.66	91.72 ± 1.13	0.92

#### Matrix Effect and Recovery

As shown in Table 2, the extraction recovery of GCs in two types of blank matrices ranged from 84.97% to 107.01%, and the SD values of the recovery were less than 6.13%. There results indicated that the simple method of sample preparation could provide an excellent extraction efficiency for all the 12 GC analytes from the solid and liquid matrices. The absolute matrix effects of all the 12 GCs were within an acceptable range from 85.84% to 109.11%, while the relative matrix effects ranged from 0.43% to 6.79%, suggesting that this assay was reliable for the analysis in the matrix.

#### Stability

The results of stability performed at three different concentrations (low, medium, and high) resulted in RSD ranging from 0.37% to 6.40%, suggesting that all GC compounds were stable in the autosampler (24 h) at 10°C (Table 2).

### Comparison With Published Methods

We compared our proposed method with methods reported in the literature for screening of GCs in varying matrices. Compared with other Q-TOF MS methods, our method has a lower LOD and wider linear range (Kim et al., 2017; Jin et al., 2018). Because of the limitations of Q-TOF MS itself, the LOD of the proposed method is not as low as that of QQQ MS and Orbitrap MS. However, a large number of synthetic drugs in dietary supplements and herbal products were illegally added to improve the short-term therapeutic effect, so our proposed method can meet the requirement of detection sensitivity. In addition, the all-ion fragmentation acquisition enabled qualitative and quantitative results in a single run, which means higher efficiency for daily work. More importantly, our CFILC method gives results of a high confidence level regarding the fast screening of illegal adulterants that are not included in the library, as well as new emerging structure analogs.

## Conclusion

In this study, a UHPLC-QTOF-MS method with AIF acquisition was established, and a new method of data processing, called CFILC, was developed for a fast screening and identification of illegal adulterated GCs in dietary supplements and herbal products. The proposed method could provide results with a high level of confidence regarding the fast screening of illegal adulterants that were not included in the library and new emerging structure analogs through a comprehensive and complete characteristic of the ions and appropriate threshold of similarity scores trained by various test samples. The CFILC method was automatically operated and expandable, and a customizable design according to the study needs was provided and enabled the use of other classes of illegal adulterants with similar structure. The obtained validation results and real samples demonstrated the great qualitative and quantitative strength of this newly developed UHPLC-QTOF-MS method with AIF acquisition and CFILC method. In conclusion, UHPLC-QTOF-MS combined with the AIF acquisition and CFILC method was a powerful tool for a fast screening and identification of illegal adulterants in dietary supplements and herbal products.

## Data Availability

The original contributions presented in the study are included in the article/Supplementary Material; further inquiries can be directed to the corresponding authors.

## References

[B1] Barnes P. J. Adcock I. M. (2009). Glucocorticoid Resistance in Inflammatory Diseases. The Lancet 373, 1905–1917. 10.1016/s0140-6736(09)60326-3 19482216

[B2] Delahaut P. Jacquemin P. Colemonts Y. Dubois M. De Graeve J. Deluyker H. (1997). Quantitative Determination of Several Synthetic Corticosteriods by Gas Chromatography-Mass Spectrometry after Purification by Immunoaffinity Chromatography. J. Chromatogr. B: Biomed. Sci. Appl. 696, 203–215. 10.1016/s0378-4347(97)00222-3 9323541

[B3] Fung F. Linn Y. (2017). Steroids in Traditional Chinese Medicine: what Is the Evidence? smedj 58, 115–120. 10.11622/smedj.2017016 PMC5360864 28361161

[B4] Geiger T. Cox J. Mann M. (2010). Proteomics on an Orbitrap Benchtop Mass Spectrometer Using All-Ion Fragmentation. Mol. Cell Proteomics 9, 2252–2261. 10.1074/mcp.m110.001537 20610777 PMC2953918

[B5] Gillet L. C. Navarro P. Tate S. Röst H. Selevsek N. Reiter L. (2012). Targeted Data Extraction of the MS/MS Spectra Generated by Data-independent Acquisition: a New Concept for Consistent and Accurate Proteome Analysis. Mol. Cell Proteomics 11, O111.016717. 10.1074/mcp.O111.016717 PMC3433915 22261725

[B6] Golubovic J. B. Otasevic B. M. Protic A. D. Stankovic A. M. Zecevic M. L. (2015). Liquid Chromatography/tandem Mass Spectrometry for Simultaneous Determination of Undeclared Corticosteroids in Cosmetic Creams. Rapid Commun. Mass. Spectrom. 29, 2319–2327. 10.1002/rcm.7403 26563702

[B7] Haneef J. Shaharyar M. Husain A. Rashid M. Mishra R. Parveen S. (2013). Application of LC-MS/MS for Quantitative Analysis of Glucocorticoids and Stimulants in Biological Fluids. J. Pharm. Anal. 3, 341–348. 10.1016/j.jpha.2013.03.005 29403837 PMC5760999

[B8] Huérfano Barco I. M. España Amórtegui J. C. Guerrero Dallos J. A. (2022). Development and Validation of Qualitative Screening, Quantitative Determination and post-targeted Pesticide Analysis in Tropical Fruits and Vegetables by LC-HRMS. Food Chem. 367, 130714. 10.1016/j.foodchem.2021.130714 34388632

[B9] Jin P. Liang X. Wu X. He X. Kuang Y. Hu X. (2018). Screening and Quantification of 18 Glucocorticoid Adulterants from Herbal Pharmaceuticals and Health Foods by HPLC and Confirmed by LC-Q-TOF-MS/MS. Food Additives & Contaminants: A 35, 10–19. 10.1080/19440049.2017.1400184 29095118

[B10] Ki N.-Y. Hur J. Kim B. H. Kim K. H. Moon B. J. Oh H. B. (2019). Rapid Screening of Sulfonamides in Dietary Supplements Based on Extracted Common Ion Chromatogram and Neutral Loss Scan by LC-Q/TOF-mass Spectrometry. J. Food Drug Anal. 27, 164–174. 10.1016/j.jfda.2018.08.006 30648569 PMC9298626

[B11] Kim E. H. Seo H. S. Ki N. Y. Park N.-H. Lee W. Do J. A. (2017). Reliable Screening and Confirmation of 156 Multi-Class Illegal Adulterants in Dietary Supplements Based on Extracted Common Ion Chromatograms by Ultra-high-performance Liquid Chromatography-Quadrupole/time of Flight-Mass Spectrometry. J. Chromatogr. A 1491, 43–56. 10.1016/j.chroma.2017.02.032 28238425

[B12] Laforest S. Pelletier M. Denver N. Poirier B. Nguyen S. Walker B. R. (2019). Simultaneous Quantification of Estrogens and Glucocorticoids in Human Adipose Tissue by Liquid-Chromatography-Tandem Mass Spectrometry. J. Steroid Biochem. Mol. Biol. 195, 105476. 10.1016/j.jsbmb.2019.105476 31561001 PMC7099401

[B13] Li J. Cui Y. Liu D. Li M. Gao J. Ye J. (2021). Development of a Sample Pretreatment Device Integrating Ultrasonication, Centrifugation and Ultrafiltration, its Application on Rapid On-Site Screening of Illegally Added Chemical Components in Heat-Clearing, Detoxicating Chinese Patent Medicines Followed by Electrospray Ionization-Ion Mobility Spectrometry. J. Pharm. Biomed. Anal. 194, 113767. 10.1016/j.jpba.2020.113767 33279301

[B14] Luque-Córdoba D. Priego-Capote F. (2021). Fully Automated Method for Quantitative Determination of Steroids in Serum: An Approach to Evaluate Steroidogenesis. Talanta 224, 121923. 10.1016/j.talanta.2020.121923 33379124

[B15] Park H.-J. Cho S.-H. Lee J. H. Hwang I. S. Han K. M. Yoon C.-Y. (2016). Screening for Corticosteroid Adulterants in Korean Herbal Medicines. J. Forensic Sci. 61, 226–229. 10.1111/1556-4029.12906 26346959

[B16] Protti M. Mandrioli R. Mercolini L. (2020). Microsampling and LC-MS/MS for Antidoping Testing of Glucocorticoids in Urine. Bioanalysis 12, 769–782. 10.4155/bio-2020-0044 32530296

[B17] Reig M. Mora L. Navarro J. L. Toldrá F. (2006). A Chromatography Method for the Screening and Confirmatory Detection of Dexamethasone. Meat Sci. 74, 676–680. 10.1016/j.meatsci.2006.05.018 22063222

[B18] Schacke H. Docke W. D. Asadullah K. (2002). Mechanisms Involved in the Side Effects of Glucocorticoids. Pharmacol. Ther. 96, 23–43. 10.1016/s0163-7258(02)00297-8 12441176

[B19] Thoren K. L. Colby J. M. Shugarts S. B. Wu A. H. B. Lynch K. L. (2016). Comparison of Information-dependent Acquisition on a Tandem Quadrupole TOF vs a Triple Quadrupole Linear Ion Trap Mass Spectrometer for Broad-Spectrum Drug Screening. Clin. Chem. 62, 170–178. 10.1373/clinchem.2015.241315 26453698

[B20] Tsugawa H. Cajka T. Kind T. Ma Y. Higgins B. Ikeda K. (2015). MS-DIAL: Data-independent MS/MS Deconvolution for Comprehensive Metabolome Analysis. Nat. Methods 12, 523–526. 10.1038/nmeth.3393 25938372 PMC4449330

[B21] Tsugawa H. Kind T. Nakabayashi R. Yukihira D. Tanaka W. Cajka T. (2016). Hydrogen Rearrangement Rules: Computational MS/MS Fragmentation and Structure Elucidation Using MS-FINDER Software. Anal. Chem. 88, 7946–7958. 10.1021/acs.analchem.6b00770 27419259 PMC7063832

[B22] Venable J. D. Dong M.-Q. Wohlschlegel J. Dillin A. Yates J. R. (2004). Automated Approach for Quantitative Analysis of Complex Peptide Mixtures from Tandem Mass Spectra. Nat. Methods 1, 39–45. 10.1038/nmeth705 15782151

[B23] Ventura G. Bianco M. Calvano C. D. Losito I. Cataldi T. R. I. (2020). HILIC-ESI-FTMS with All Ion Fragmentation (AIF) Scans as a Tool for Fast Lipidome Investigations. Molecules 25, 2310. 10.3390/molecules25102310 PMC7287777 32423109

[B24] Wang R. Yin Y. Zhu Z.-J. (2019). Advancing Untargeted Metabolomics Using Data-independent Acquisition Mass Spectrometry Technology. Anal. Bioanal. Chem. 411, 4349–4357. 10.1007/s00216-019-01709-1 30847570

[B25] Wang Z. Wang H. Peng Y. Chen F. Zhao L. Li X. (2020). A Liquid Chromatography-Tandem Mass Spectrometry (LC-MS/MS)-based Assay to Profile 20 Plasma Steroids in Endocrine Disorders. Clin. Chem. Lab. Med. (Cclm) 58, 1477–1487. 10.1515/cclm-2019-0869 32084000

[B26] Wasito H. Causon T. Hann S. (2022). Alternating In-Source Fragmentation with Single-Stage High-Resolution Mass Spectrometry with High Annotation Confidence in Non-targeted Metabolomics. Talanta 236, 122828. 10.1016/j.talanta.2021.122828 34635218

[B27] Whitman J. D. Lynch K. L. (2019). Optimization and Comparison of Information-dependent Acquisition (IDA) to Sequential Window Acquisition of All Theoretical Fragment Ion Spectra (SWATH) for High-Resolution Mass Spectrometry in Clinical Toxicology. Clin. Chem. 65, 862–870. 10.1373/clinchem.2018.300756 30996055

[B28] Zhou S. Guo C. Shi F. Jiang W. Wang L. (2016). Application of an Ultrahigh-Performance Liquid Chromatography Coupled to Quadrupole-Orbitrap High-Resolution Mass Spectrometry for the Rapid Screening, Identification and Quantification of Illegal Adulterated Glucocorticoids in Herbal Medicines. J. Chromatogr. B 1038, 34–42. 10.1016/j.jchromb.2016.10.010 27783980

