# Supplementary material for: Fast Screening and Identification of Illegal Adulterated Glucocorticoids in Dietary Supplements and Herbal Products Using UHPLC-QTOF-MS With All-Ion Fragmentation Acquisition Combined With Characteristic Fragment Ion List Classification
Source: Front Chem. 2021 Dec 10;9:785475. doi: 10.3389/fchem.2021.785475 (PMC8702623; doi:10.3389/fchem.2021.785475)
Supplement: Supplementary file 1 [file DataSheet1.PDF]

Fast screening and identification of illegal adulterated glucocorticoids in dietary supplements and herbal products using UHPLC-QTOF-MS with all ion fragmentation acquisition combined with characteristic fragment ion list classification

Ying Xue[a,b, 1], Yanghao Sheng [a,b, 1], Jue Wang [a,b], Qi Huang [a,b], Fengyu Zhang [a], Ying Wen [a], Shao Liu[a,b, *], Yueping Jiang [a,b, *]

[a] *Department of Pharmacy, Xiangya Hospital, Central South University, Changsha 410008, China*

[b] *Institute for Rational and Safe Medication Practices, National Clinical Research Center for Geriatric Disorders, Xiangya Hospital, Central South University, Changsha 410008, China*

Supplementary information

[*] Corresponding authors.

E-mail address: liushao999@csu.edu.cn (S. Liu); jiangyueping@csu.edu.cn (Y. Jiang)

[1] These authors contributed equally to this article.

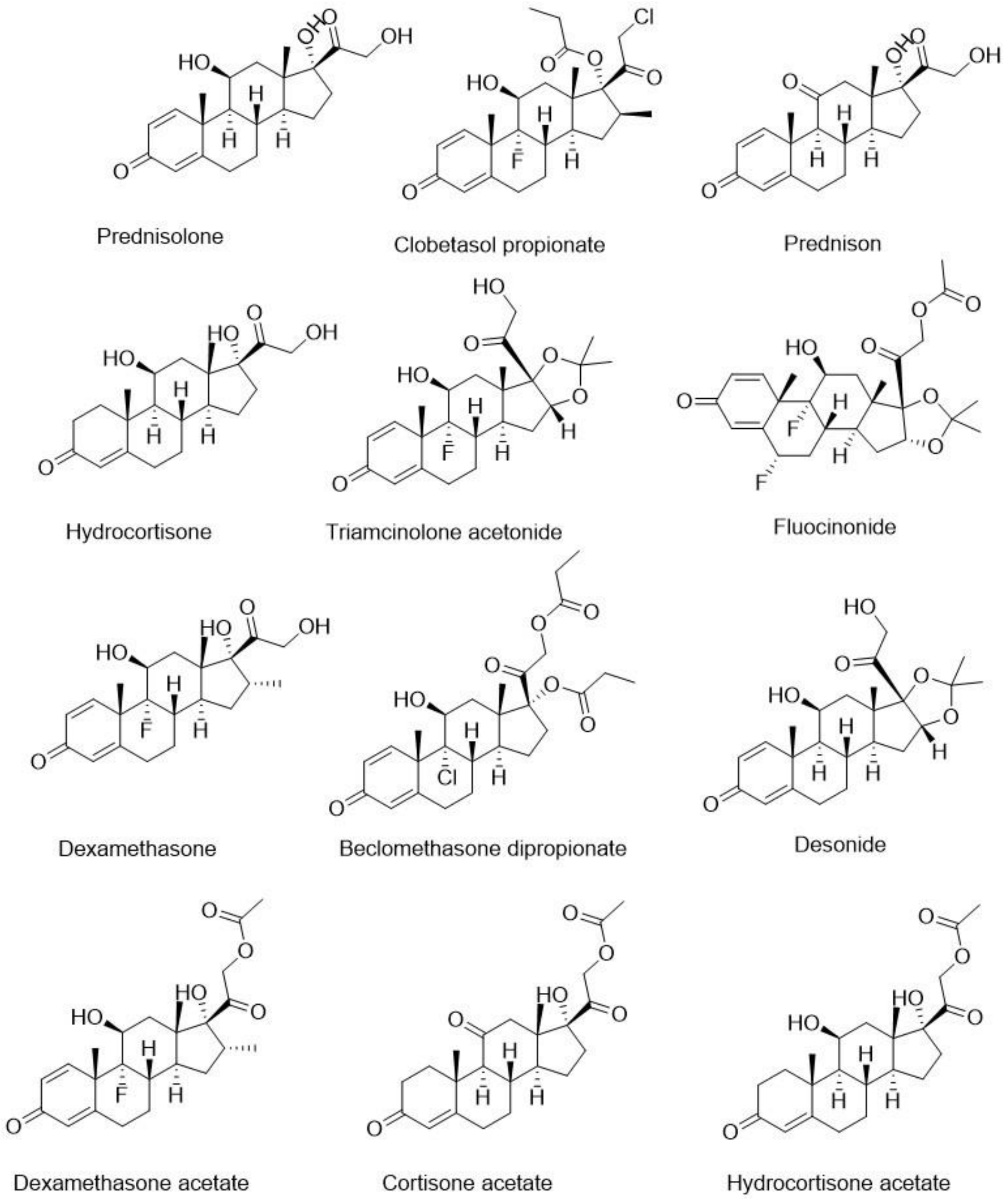

Figure S1. The structures of 12 investigated glucocorticoids.

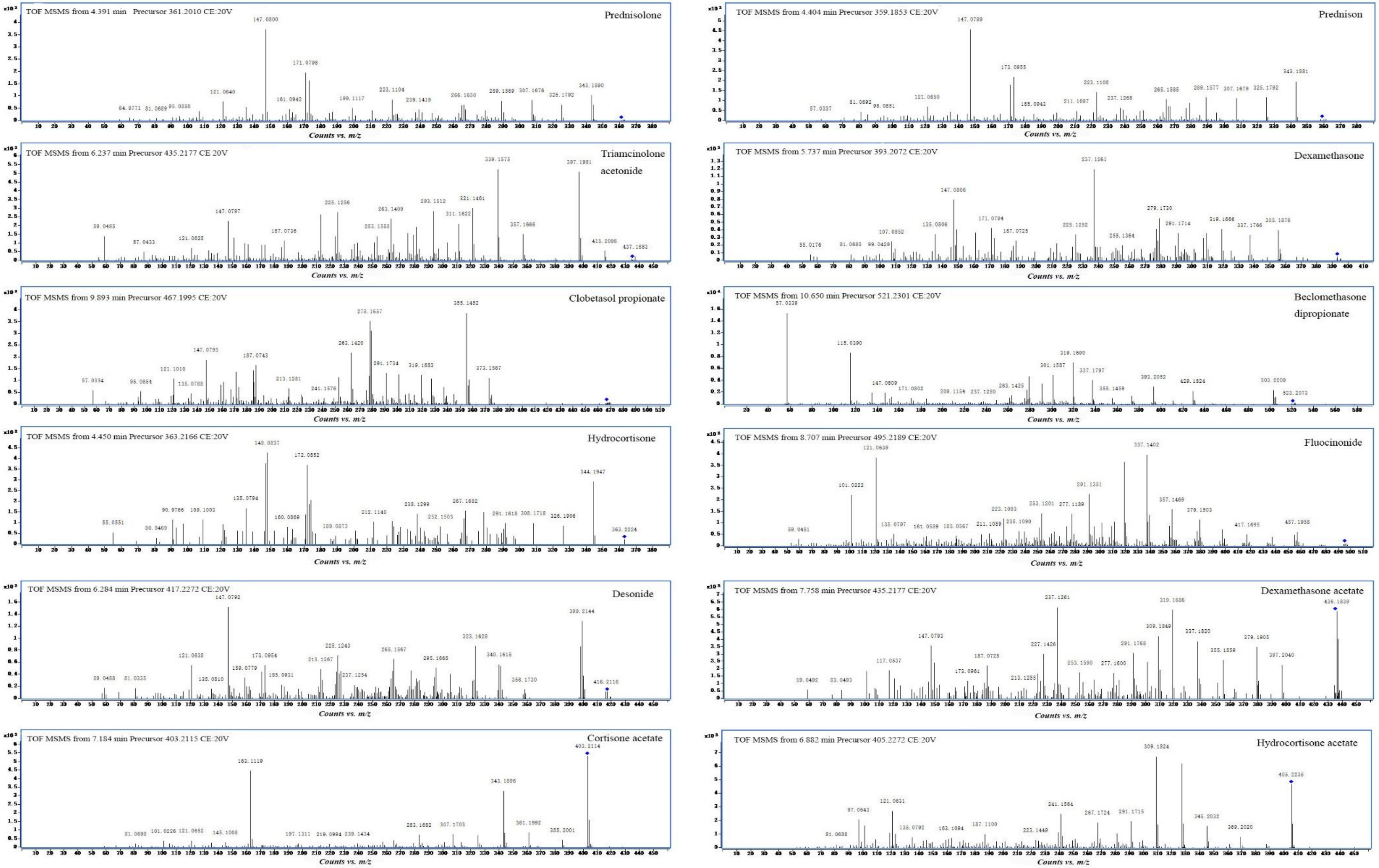

Figure S2. MS^2 spectra for 12 investigated glucocorticoids at collision energy of 20 eV.

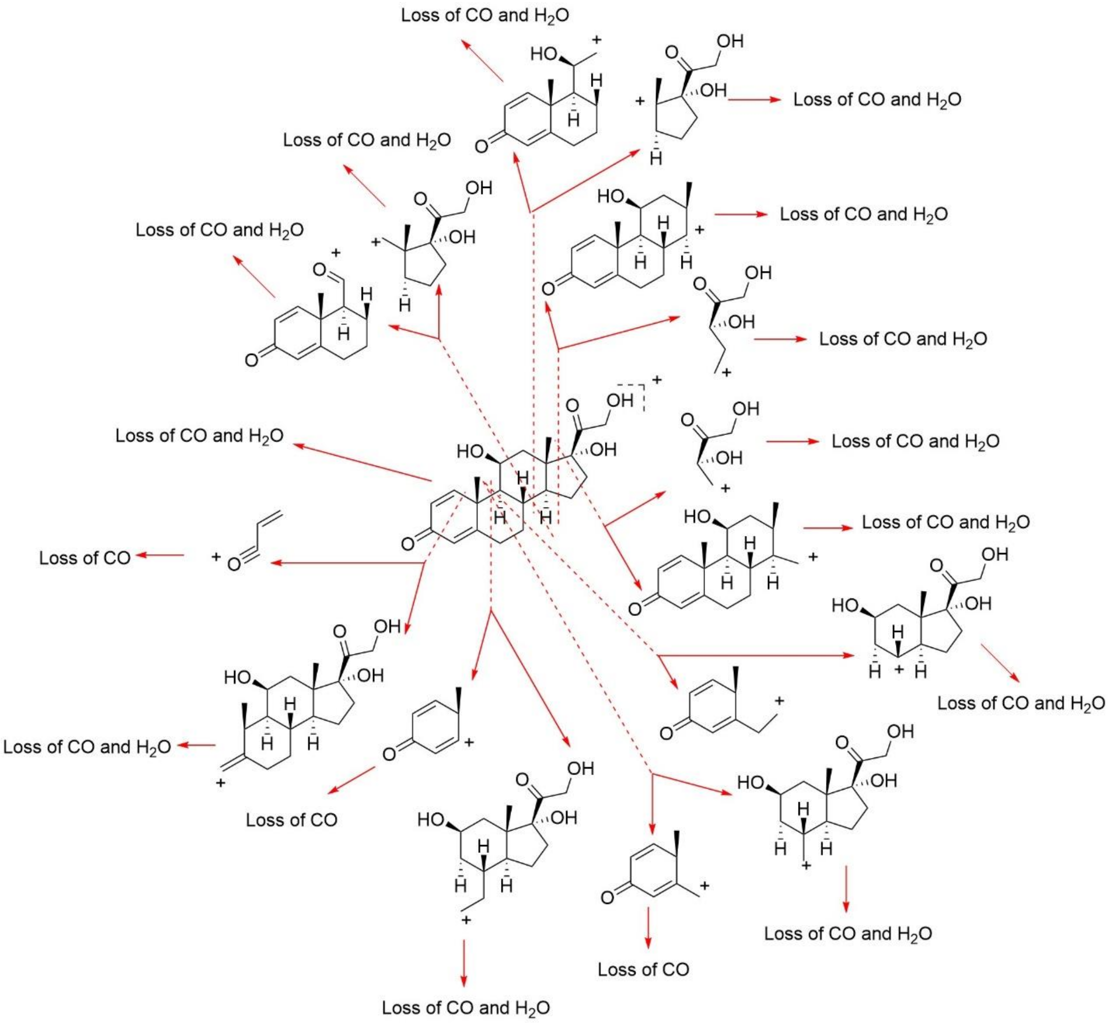

Figure S3. The fragmentation pattern of one representative glucocorticoid (prednisolone).

Table S1. Parameters of MS-DIAL

#Data collection parameters	
Retention time begin	0
Retention time end	16
Mass range begin	0
Mass range end	2000
MS2 mass range begin	0
MS2 mass range end	2000
#Centroid parameters	
MS1 tolerance	0.01
MS2 tolerance	0.025
#Peak detection parameters	
Smoothing method	Linear Weighted Moving Average
Smoothing level	3
Minimum peak width	5
Minimum peak height	3000
#Peak spotting parameters	
Mass slice width	0.1
#Deconvolution parameters	
Sigma window value	0.8
MS2Dec amplitude cut off	100
Exclude after precursor	True
Keep isotope until	0.5
Keep original precursor isotopes	False
